# Mechanochemical Functionalization of Oxidized Carbon Black with PLA

**DOI:** 10.3390/molecules30010094

**Published:** 2024-12-29

**Authors:** Aida Kiani, Mattia Naddeo, Federica Santulli, Valentina Volpe, Mina Mazzeo, Maria Rosaria Acocella

**Affiliations:** 1 Dipartimento di Chimica e Biologia “A. Zambelli”, Università degli Studi di Salerno, Via Giovanni Paolo II, I-84084 Fisciano, SA, Italy; m.naddeo21@studenti.unisa.it (M.N.); fsantulli@unisa.it (F.S.); mmazzeo@unisa.it (M.M.); 2 Dipartimento di Ingegneria Industriale, Università degli Studi di Salerno, Via Giovanni Paolo II, I-84084 Fisciano, SA, Italy; vavolpe@unisa.it

**Keywords:** functionalization, mechanochemistry, carbon black, waste recovery, PLA

## Abstract

The functionalization of carbon black (CB) represents a promising strategy to enhance its compatibility with polymers while addressing sustainability concerns. In this study, a solvent-free mechanochemical approach (ball milling) is proposed for the functionalization of oxidized carbon black (oCB) with post-consumed polylactic acid (PLA), overcoming the environmental drawbacks of conventional methods that mostly rely on toxic solvents and catalysts. The functionalized carbon black (f-CB) was characterized by Fourier transform infrared spectroscopy (FTIR), elemental analysis (EA), and thermogravimetric analysis (TGA) to confirm the successful modification. At the same time, the influence of f-CB as a nanofiller of residual PLA waste was evaluated using differential scanning calorimetry (DSC) and gel permeation chromatography (GPC), demonstrating its stabilization effect during melt extrusion by preserving the molecular weight of the starting polymer. On the other hand, the dynamic mechanical analysis (DMA) revealed that the addition of f-CB did not negatively affect the mechanical properties of the resulting composite. In conclusion, mechanochemistry was used as a sustainable and unique strategy for the upcycling of waste PLA into a PLA-based composite stabilized by CB functionalized with the waste PLA itself.

## 1. Introduction

Polymeric materials have become indispensable in daily life due to their light weight, low cost, and high durability [1,2]. However, their longevity represents a critical issue since their end-of-life treatment is still not well planned, and, after consumption, they are usually stored in the landfill or accumulate in the environment. Aliphatic polyesters, as bio-based plastics, emerge as potential substitutes for petrochemical-based polymers, attracting increasing attention due to their perceived sustainability and circularity. Among these, polylactic acid (PLA), thanks to its good processability, as well as excellent strength and modulus, is the most prominent bio-based plastic. Despite its advantages, PLA’s slow degradation in natural environments may contribute to persistent plastic pollution issues [3,4,5,6]. Addressing these environmental concerns, plastic recycling has emerged as a crucial strategy and is generally categorized into mechanical recycling and chemical recycling [7,8,9]. Although aliphatic polyesters like PLA contain chemically active ester groups, making chemical recycling a promising strategy for breaking down polymers into monomers or building blocks for repolymerization, the complexity and high costs associated with conventional chemical recycling methods, which often require homogeneous conditions and activating reagents, have limited their widespread adoption [10,11,12,13]. Therefore, mechanically recycling the PLA waste could be an interesting opportunity in terms of sustainability. This method is well consolidated for the recovery of post-consumer polyethylene terephatalate but was revealed to be an ineffective approach for aliphatic polyesters. In fact, PLA degradation, even of the virgin polymer, is particularly pronounced during melt processing, typically at around 200 °C, which often results in a reduced molecular weight [14,15]; on the other hand, PLA can exhibit poor mechanical properties, particularly above its glass transition temperature (Tg ≈ 60 °C), due to its slow crystallization rate, which often results in low crystallinity. As a result, to stabilize PLA during melt processing and to promote faster crystallization, several approaches have been proposed. These include compounding with antioxidants, water scavengers, and chain extenders such as organic phosphites and functional polysilsesquioxane microspheres [16,17]; or nanofillers like clays and organoclays [18], silica [19], lignin [20] and silk [21]; as well as graphene [22,23], high-surface-area graphite [24], and carbon black [25,26,27,28]. Recent studies of alkylated carbon black (aCB) showed the double ability of the filler to act as a nucleating agent for PLA and as a molecular weight stabilizer. The presence of long alkyl chains attached to graphitic layers promotes the nucleation of PLA crystallization possibly due to the formation of ordered local aggregates on solid structural layers, serving as effective crystallization nuclei for the linear aliphatic polyester. On the other hand, the carbon filler can act as a scavenger of water, preventing degradation induced by hydrolysis [29]. Recently, bio-based conductive polymer composites with excellent electrical conductivity were produced by a scalable strategy of an “annealing–stretching–thermal treatment” [30] of PLA with CB.

In this work, we propose a new strategy for the functionalization of CB with PLA via mechanochemistry. For decades, mechanochemical degradation in polymers was largely confined to random chain scission. However, recent advancements have revealed the potential for mechanochemical recycling that offers more controlled and targeted approaches for polymer breakdown and recovery. These developments have been applied to various commercial polymers, including both thermoplastics and thermosets [31,32]. It is well known that mechanochemistry [33], and specifically the ball milling approach, provides a sustainable and cost-effective alternative for polymer recycling [32,34,35]. Indeed, during the milling process, the collision and shear forces generated between the balls, or balls and the vessel walls, are transmitted to the polymer chains, potentially inducing chemical transformations [34,36,37]. Recent advancements in polymer mechanochemistry highlight the potential of using mechanical forces to induce constructive chemical transformations, presenting a unique route for polymer degradation and recycling. By eliminating solvents, these methods reduce costs and environmental impacts, and align with green chemistry principles, offering improved atom economy, safer processes, and minimal waste [38,39,40]. It has been demonstrated that applying mechanical stress to polymeric materials can lead to degradation, resulting in particularly interesting polymers with the tendency to easily degrade, such as polylactic acid (PLA), polyglycolic acid (PGA), polycaprolactone (PCL), polyhydroxybutyrate (PHB), etc. [41]. In this context, the present study proposes the use of mechanochemistry to enhance sustainability and cost-effectiveness techniques to effectively functionalize oCB with PLA, without the need for solvents or catalysts, as the functionalization has already been performed in solution in the presence of a toxic solvent. Therefore, an alternative sustainable procedure that makes possible the functionalization in solvent-free conditions could be interesting for meeting the environmental requests. Additionally, ball milling technology could fruitfully provide mechanical energy and contact that can facilitate the functionalization of the carbon material. Exploiting the PLA waste as a reagent to promote a transesterification reaction with the oCB could represent a promising approach in an optic of a closed loop for the upcycling of PLA. This study will also investigate the effect of small amounts of functionalized carbon black (f-CB), prepared through ball milling, as a nanofiller of the residual PLA waste to evaluate the possible stabilization during processing and the enhancement of the mechanical properties of the resulting composite.

## 2. Results

### 2.1. Functionalization of oCB with HM_m_ PLA and LM_m_ PLA

**Figure 1:**
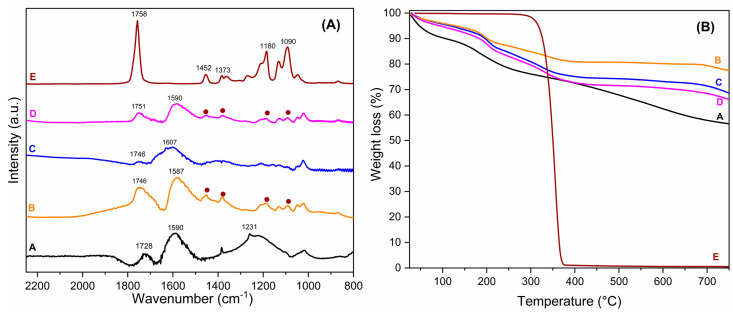
(**A**) FTIR spectra and (**B**) TGA scans of the oCB before and after functionalization with HM_m_ PLA at different times with the assistance of H_2_O. oCB (A); f-CB at 60 min (B); f-CB at 120 min (C); f-CB1 at 180 min (D); and HM_m_ PLA (E). Solid dots showing the wavenumbers related to PLA.

A commercial carbon black sample, exhibiting a BET surface area of 130 m^2^ g^−1^, has been oxidized by the Hummers method, which was chosen as the oxidation method for providing suitable oxygen functionalities for further modification. As evaluated by the elemental analysis, oxidized carbon black (oCB) was obtained with 0.44 as the O/C weight ratio. As reported in FTIR (Figure 1A), the typical vibrations between 1000 and 1300 cm^−1^ due to alcohols, ethers, and vinyl ethers and the absorption band at 1590 cm^−1^ due to the vibrations associated with the double bonds of carbon, and finally one at 1728 cm^−1^ relating to the carbonyl group are evident in the spectrum of oCB, as well as the degradation associated with the oxygenated functionalities in TGA (Figure 1B) [42].

**Figure 2:**
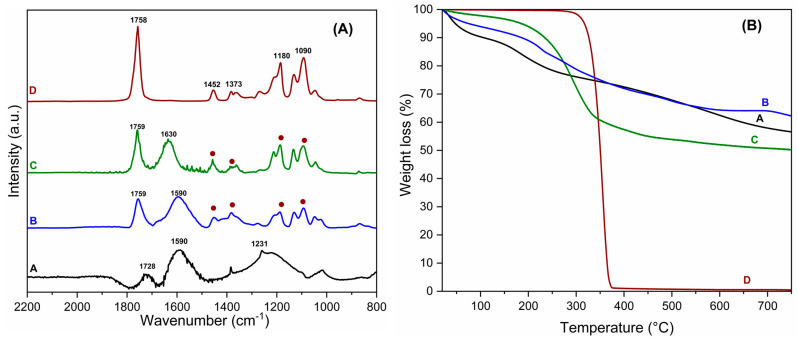
(**A**) FTIR spectra and (**B**) TGA scans of the oCB before and after functionalization at 180 min of ball milling with LM_m_ PLA with the assistance of H_2_O and methanol. oCB (A), f-CB with the assistance of H_2_O (B), f-CB with the assistance of methanol (C), and LM_m_ PLA (D). Solid dots showing the wavenumbers related to PLA.

PLA was also characterized by FTIR, TGA, and GPC analyses to confirm the nature and molecular mass of the polymer. As reported in Figure 2A, PLA shows a characteristic stretching peak at 1758 cm^−1^ related to the carbonyl groups (–C=O), a peak at 1180 cm^−1^ ascribed to –C–O– stretching vibration, as well as other peaks at 1452, 1373, and 1090 cm^−1^ that refer to the signals from the alkyl chains of the polymer [43].

**Scheme 1:**
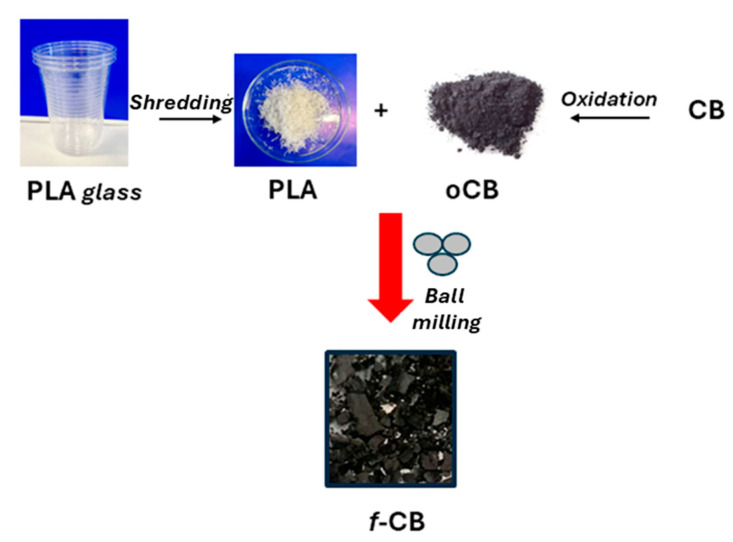
Representative scheme to illustrate the stages of the nanofiller synthesis.

The functionalized carbon black (f-CB1) powders were obtained following the steps showed in Scheme 1 by varying the ball milling reaction times (60 min, 120 min, and 180 min) in the presence of high-molecular-mass (HM_m_) PLA (55 kDa) and 0.1 mL of water, as confirmed by the FTIR analysis.

Specifically, the spectra revealed a shift in the carboxylic acid peak of oCB, indicating the formation of ester groups, with a change from 1728 cm^−1^ to 1751 cm^−1^. Furthermore, characteristic PLA vibrations were observed in the f-CB, with peaks at 1450, 1371, 1180, and 1091 cm^−1^, demonstrating the successful interaction between PLA and carbon black.

A significantly different degradation profile was observed for all f-CB adducts by TGA compared to the oCB and PLA starting materials. Both the FTIR analysis and TGA confirmed the ability of PLA with a high molecular mass to functionalize oCB under ball milling reaction conditions. This conversion could occur due to covalent-type mechanochemical functionalization with the assistance of a negligible amount of solvent (H_2_O). In mechanochemical processes, using only solid materials and adding small amounts of liquid through liquid-assisted grinding (LAG) can significantly enhance the reaction yield and speed [44]. The reaction conducted for 180 min appeared to be optimal, achieving the desired adduct (f-CB_1_) with a high mass yield of 99%.

To better understand the effect of PLA on altering the characteristics of oCB, a similar reaction was conducted using low-molecular-mass PLA (LM_m_ = 9 kDa) under ball milling for 180 min.

The FTIR and TGA results (Figure 2A,B) revealed that LM_m_ PLA in the presence of water provided a higher degree of functionalization for oCB compared to HM_m_ PLA, as shown in Figure 1A,B), D profile. This increased functionalization is likely due to the shorter and more exposed polymer chains that can easily react with the carbonaceous material’s surface. To determine the impact of the solvent, methanol was tested under the same reaction conditions, and it was found to facilitate the reaction more effectively than water, providing f-CB_2_ as the best sample. This is evidenced by the more pronounced PLA signals observed in the FTIR spectra, as well as the TGA profile, when methanol is used. The ability of methanol to interact more readily with the polymer chains and possibly lead to swelling of the polymer matrix and enhanced chain mobility could be responsible for higher functionalization.

### 2.2. Effect of f-CB on Recycled PLA Processing

Carbon black can be used as reinforcement in PLA to address its brittleness and low thermal deformation resistance and, when conveniently functionalized, can accelerate the crystallization rate of PLA [43,45,46]. Additionally, can be added as filler during melt extrusion, preserving the molecular weight of PLA [24].

Conversely, oCB is not a useful filler due to the presence of hydroxyl and carboxyl groups that can promote the hydrolytic degradation of PLA during the processing.

Therefore, further proof of the successful mechanical functionalization of carbon black (f-CB) can be found in the realization of the PLA compound by melt processing.

This study was conducted in the presence of functionalized carbon black (f-CB) with both low and high molecular masses of PLA as a filler to evaluate not only the workability but also a potential improvement of the mechanical properties of recycled PLA. In order to investigate these aspects, an f-CB/PLA composite was produced using an extruder, including the first 1% by mass of f-CB_1_ that was obtained from a 180 min ball milling with high-molecular-mass PLA and water and then the one obtained from the reaction with methanol and low-molecular-mass PLA (f-CB_2_). The resulting composites were checked by GPC and DSC. To evaluate the real processability conditions, extrusion was performed at 170 °C, and two different batches of the same PLA brand were used.

Interestingly, the GPC results (Table 1) showed that the molecular masses of the PLA and the composite remained almost unchanged after the mixing, thus highlighting the stability of the filler within the composite, which did not lead to any degradation phenomena of the PLA and confirming the successful mechanochemical functionalization of oCB (Table 1, Entries 2–4).

DSC analysis was used to determine the effect of f-CB_1_ on the thermal transitions of processed PLA composites. The first heating cycle was performed to eliminate the thermal history of the polymeric material [47]. As a result, the thermal behavior of the second heating cycle was considered for discussion. The glass transition temperature (T_g_), melting temperature (T_m_), cold crystallization temperature (T_cc_), experimental melting enthalpy (ΔH_m_), and cold crystallization enthalpy (ΔH_cc_) of neat samples and PLA composites are reported in Table 2.

**Figure 3:**
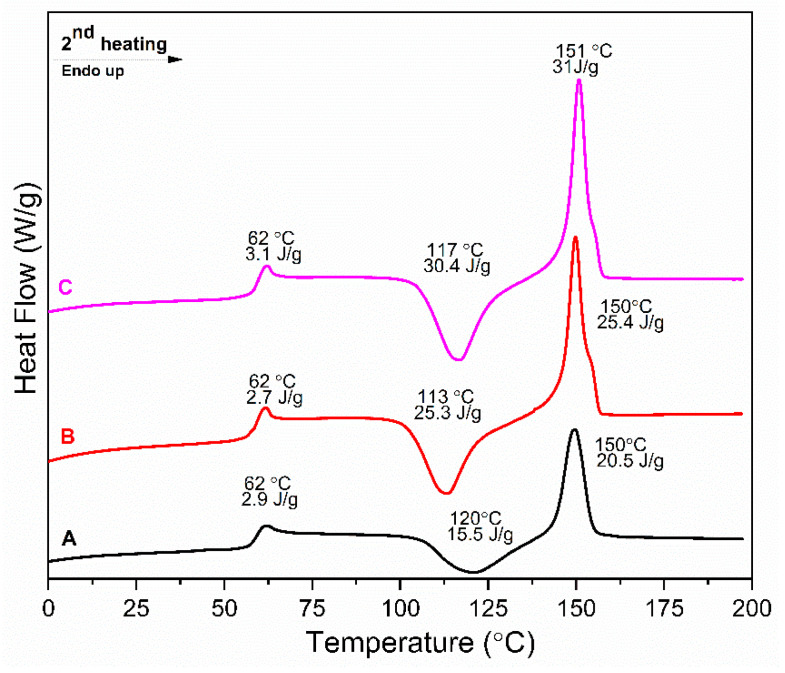
DSC scans of the HM_m_ PLA (first batch) before (A) and after (B) extrusion and a composite of f-CB1 (obtained from 180 min of ball milling with HM_m_ PLA and water) with HM_m_ PLA (first batch) after extrusion at 170 °C (C).

As evidenced in Table 2 and Figure 3, contrary to what has been observed in our previous work [29], no nucleation effect was observed by adding f-CB_1_. This could be possibly due to an easier oCB attack of the side chain of the polymer, providing a reduced alkyl chain length on the carbon surface. The PLA, T_g_, as well as the ΔH_m_ and ΔH_cc_, are essentially the same after extrusion with or without adding f-CB_1_, showing that no negative effect can be exerted by the functionalized filler.

**Figure 4:**
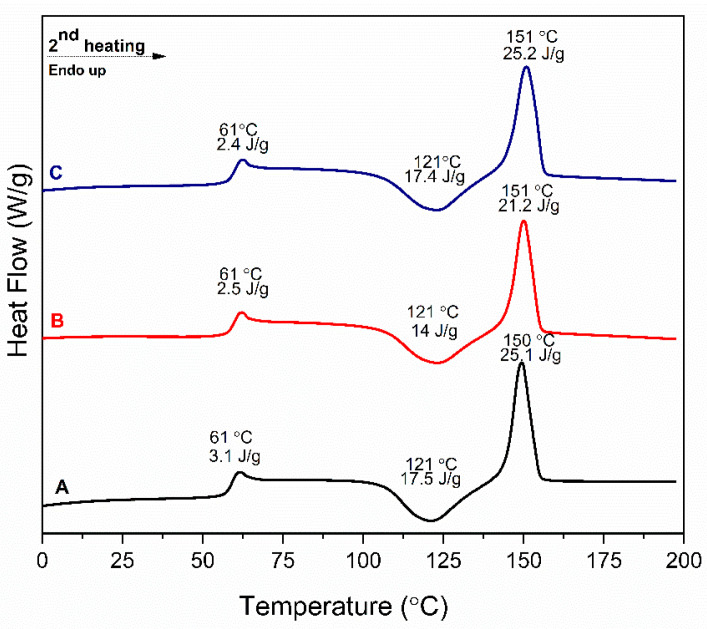
DSC scans of the HM_m_ PLA (second batch) before (A) and after (B) extrusion and a composite of f-CB_2_ (obtained from 180 min of ball milling with LM_m_ PLA and ethanol) with processed PLA (second batch) after extrusion at 170 °C (C).

To evaluate whether the degree of functionalization could influence the increase in the processability and possibly the crystallization ability of the composite, a further study was carried out using 1% f-CB_2_ obtained from the reaction with methanol and low-molecular-mass PLA (Figure 4).

To show the general scope of the procedure, a second batch of the same brand was used as recycled PLA.

The GPC results before and after extrusion showed a consistent stabilization effect on the PLA composite exerted by the f-CB_2_ during melt extrusion, preserving the molecular weight of the starting polymer (Table 1, Entries 5–7).

Although the degree of functionalization obtained for f-CB_2_ was higher than f-CB_1_ (see Figure 2B vs. Figure 1B), again, no nucleation effect was observed after processing, leaving the degree of crystallization substantially unaltered.

### 2.3. Mechanical Property Investigation

**Figure 5:**
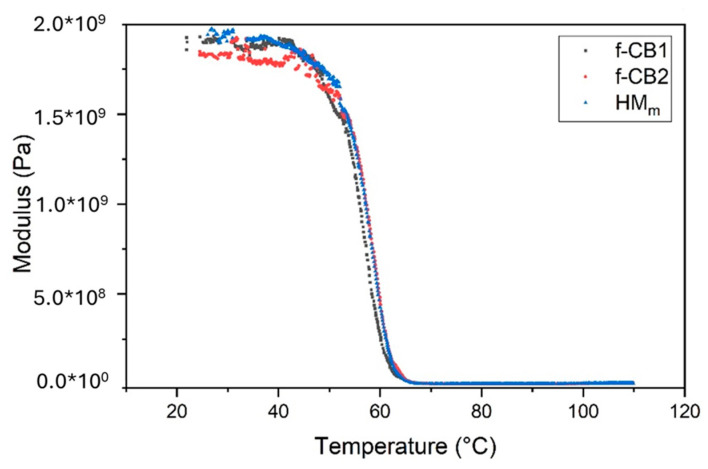
Evolution of the modulus with the temperature for HM_m_ PLA, f-CB_1_, and f-CB_2_.

**Figure 6:**
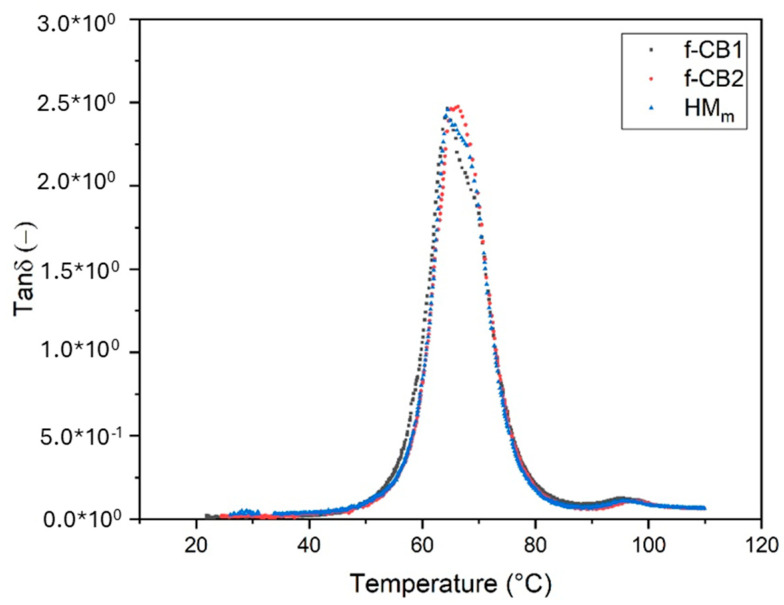
Evolution of the tanδ with the temperature for HM_m_ PLA, f-CB_1_, and f-CB_2_.

The DMA analysis in tension mode allowed the investigation of the mechanical properties of HM_m_ PLA, f-CB_1_, and f-CB_2_. Figure 5 and Figure 6 show, respectively, the evolution of the modulus and tanδ with the temperature.

The modulus of HM_m_ PLA at low temperatures was about 1.9 × 10^9^ Pa and underwent a drastic reduction between 50 and 60 °C. The DMA results showed that the functionalized carbon black did not alter the mechanical behavior of the PLA, further confirming the mechanochemical functionalization of oCB. At low temperatures, the modulus of f-CB_1_ was almost the same as HM_m_ PLA, while the modulus of f-CB_2_ was reduced by approximately 7%. Figure 6 shows that the evolution of the tanδ with temperature was almost the same for the three materials. The glass transition temperature, which can be evaluated using localized maximum within the tanδ, results had the same values in the cases of HM_m_ PLA and f-CB_1_ (64.5 °C), while for f-CB_2_ it was slightly shifted to a higher temperature (66.3 °C).

## 3. Materials and Methods

Carbon black samples (CB) of grade N110 containing 99.8% carbon and a BET surface area of 130 m^2^ g^−1^ were purchased from Cabot Company (Boston, MA, USA). Sulfuric acid, sodium nitrate, and potassium permanganate were purchased from Sigma-Aldrich (St. Louis, MO, USA). All reagents were used as received, without purification. The high-molecular-mass (HM_m_) polylactic acid (PLA) cups (Mn = 55 kDa) were purchased from local suppliers, specifically, two batches of the Selex brand, and the low-molecular-mass (LM_m_) PLA (Mn = 9 kDa) was synthesized within the laboratory facilities [48].

### 3.1. Oxidation of Carbon Black via Hummer’s Method

Oxidized carbon black (oCB) was prepared by Hummers’ method [49]; 12 mL of sulfuric acid and 0.25 g of sodium nitrate were introduced into a 1000 mL three-neck round-bottomed flask immersed in an ice bath and 0.5 g of carbon samples were added with a magnetic stirring. After obtaining a uniform dispersion, 1.5 g of potassium permanganate was added very slowly to minimize the risk of explosion. The reaction mixture was thus heated to 35 °C and stirred for 24 h. Deionized water (70 mL) was added in small amounts into the resulting black and dark green slurry for carbon black with stirring and, finally, 0.5 mL of H_2_O_2_ (30 wt%) was gradually added. The obtained sample was poured into 700 mL of deionized water and then centrifuged at 10,000 rpm for 15 min with a Neya16 centrifuge. The oCB powders were first washed twice with 10 mL of a 5 wt% HCl aqueous solution and subsequently washed with 50 mL of deionized water. Finally, the powders were dried at 60 °C for 12 h. About 0.4 g of oCB sample was obtained.

### 3.2. Functionalization of oCB with PLA via Ball Milling

Following the synthesis and characterization of oxidized carbon black, the carbonaceous materials underwent mechanochemical processing. This was followed by a functionalization reaction involving the transesterification of PLA with both high and low molecular mass variants using methanol or water as solvents. The ball milling experiments took place at room temperature in a Planetary ball mill Pulverisette 7 Premium (Fritsch GmbH, Idar-Oberstein, Germany). A total of 90 mg of oCB powder, 10 mg of PLA, and 0.1 mL of deionized water were added directly in a Steal jar of 80 mL containing 8 zirconium balls with a diameter of 10 mm. The rotational frequency was fixed at 300 rpm for 30, 60, 120, and 180 min. The quantity of solvent utilized corresponded to a 3:1 ratio in equivalents relative to the repeating unit of the PLA monomer. The product was collected and washed with dichloromethane (CH_2_Cl_2_), and the functionalized carbon black (f-CB) was recovered via precipitation, followed by centrifugation at 7000 rpm for 7 min. The resulting slurry was subsequently dried in an oven for 2 h at 50 °C.

### 3.3. Characterization

Elemental analysis (EA): The elemental analysis was performed with a Thermo FlashEA 1112 Series CHNS-O analyzer (Thermo Fisher Scientific, Schwerte, Germany).

Thermogravimetric analysis (TGA): The thermogravimetric (TG) analysis was carried out on a Q500, from 10 to 800 °C at a heating rate of 10 °C, under N_2_ flow. Weight decreases below 100 °C were used to determine the water content.

Infrared spectroscopy (FTIR): The spectra were obtained at a resolution of 2.0 cm^−1^ with an FTIR (BRUKER Vertex70, Billerica, Massachusetts, USA) spectrometer equipped with a deuterated triglycine sulfate (DTGS) detector and a KBr beam splitter using KBr pellets. The frequency scale was internally calibrated to 0.01 cm^−1^ using a He–Ne laser. The noise was reduced by signal averaging 32 scans.

Differential scanning calorimetry (DSC): The scans, as collected by TA Q2000 equipment, (New Castle, DE, USA), were used to measure the temperature and enthalpy of PLA crystallization and the stability of the obtained copolymer. The reported scans were carried out from room temperature to 130 °C, and samples remained at this temperature for 5 min; the cooling and heating steps were performed at rates of 10 °C/min from −30 to 130 °C.

Gel permeation chromatography (GPC): The measurements were conducted by an Infinity II-Agilent (Santa Clara, CA, USA) system equipped with a refractive index detector; tetrahydrofuran (THF) was used as eluent at 35 °C at a flow rate of 1.0 mL min^−1^. The calibration curve was obtained using polystyrene standards. The analyzed samples were dissolved in THF at 50 °C with a concentration of 1 mg/mL. The obtained solutions were then filtered using a Chromafil PTFE 0.45 μm filter.

Extruder: The compounds were prepared by melt compounding in a counter-rotating twin-screw micro-compounder (HAAKE MiniLab II Micro Compounder, Thermo Fisher Scientific, Schwerte, Germany). An integrated backflow channel and a bypass valve allow residence time control. The compounds were produced at a temperature of 170 °C with a screw rotation speed of 30 rpm and a recycle time of 3 min.

Dynamic mechanical analyzer (DMA): A DMA 8000 (Perkin Elmer, Milan, Italy) was used to analyze the mechanical characteristics in tension mode. The samples were heated at 2 °C/min from ambient temperature to 110 °C; a 50 μm displacement and 1 Hz frequency were set for the tensile tests.

## 4. Conclusions

In conclusion, the mechanochemical functionalization of carbon black using PLA represents a promising approach for producing sustainable and high-performance nanofillers. This study demonstrates the effectiveness of the mechanochemical functionalization of oxidized carbon black (oCB) with polylactic acid (PLA) resulting from waste recovery under solvent-free conditions, offering a sustainable and efficient pathway to enhance the compatibility of carbon black with PLA matrices. The findings highlight that the mechanical functionalization of oCB using high-molecular-mass (HM_m_) PLA and water yielded significant modifications, as evidenced by the FTIR and TGA analyses, confirming the formation of ester bonds and successful covalent attachment of PLA chains. It was also investigated those reactions conducted with low-molecular-mass (LM_m_) PLA and methanol that exhibited an even higher degree of functionalization due to the increased mobility and reactivity of shorter polymer chains.

Both HM_m_ and LM_m_ PLA composites with functionalized carbon black (f-CB_1_ and f-CB_2_) preserved the molecular weight of the recycled PLA during melt extrusion, as confirmed by the GPC analysis, indicating the stability of the compound under the processing conditions. The DSC analysis also revealed no significant nucleation effects of the addition of f-CB_1_ or f-CB_2_ to PLA, with the thermal transitions and enthalpy values remaining consistent, suggesting that functionalized carbon black does not alter the crystallization behavior of PLA composites. For both composites, the mechanical integrity of the PLA composites was maintained. Although f-CB_2_ slightly reduced the modulus at low temperatures and slightly increased the glass transition temperature (T_g_), these effects were minimal and did not compromise the material’s performance. All this evidence highlights the stability of the filler within the composite, which does not lead to any degradation phenomena on the PLA and confirms the successful mechanochemical functionalization of oCB.

Functionalization by the ball milling approach contributes to the advancement of green chemistry and circular economy strategies by integrating waste PLA and carbon black into a closed-loop system, enhancing both environmental sustainability and material performance.

## Figures and Tables

**Table 1:** GPC results.

Entry	Sample	Mn ^a^ (kDa)	Mw ^a^ (kDa)	Ð ^a^
1	LM_m_ PLA	9.7	10	1.1
2	HM_m_ PLA (first batch) before extrusion	51	96	1.9
3	HM_m_ PLA (first batch) after extrusion	52	96	1.9
4	HM_m_ PLA (first batch)/f-CB_1_ composite	50	94	1.9
5	HM_m_ PLA (second batch) before extrusion	51	96	1.9
6	HM_m_ PLA (second batch) after extrusion	59	102	1.7
7	HM_m_ PLA (second batch)/f-CB_2_ composite	60	102	1.7

^a^ The experimental Mn, Mw, and Ð were determined by a GPC analysis in THF using a PS standard and were corrected by the factor 0.58.

**Table 2:** DSC results.

Entry	Sample	T_g_ (°C)	T_cc_ (°C)	T_m_ (°C)	ΔH_cc_ (J/g)	ΔH_m_ (J/g)
1	HM_m_ PLA (first batch) before extrusion	62	120	150	15.5	20.5
2	HM_m_ PLA (first batch) after extrusion at 170 °C	62	113	150	25.3	25.4
3	Composite of f-CB1 with processed PLA (first batch) after extrusion at 170 °C	62	117	151	30.4	31
4	HM_m_ PLA (second batch) before extrusion	61	121	150	17.5	25.1
5	HM_m_ PLA (second batch) after extrusion at 170 °C	61	121	151	14	21.2
6	Composite of f-CB2 with processed PLA (second batch) after extrusion at 170 °C	61	121	151	17.4	25.2

## Data Availability

Data are contained within the article.
